# Microwave-assisted preparation, structural characterization, lipophilicity, and anti-cancer assay of some hydroxycoumarin derivatives

**DOI:** 10.1007/s00706-014-1320-8

**Published:** 2014-10-24

**Authors:** Kinga Ostrowska, Elżbieta Hejchman, Dorota Maciejewska, Agata Włodarczyk, Kamil Wojnicki, Dariusz Matosiuk, Agnieszka Czajkowska, Izabela Młynarczuk-Biały, Łukasz Dobrzycki

**Affiliations:** 1 Department of Organic Chemistry, Faculty of Pharmacy, Medical University of Warsaw, 1 Banacha, 02 097 Warsaw, Poland; 2 Department of Synthesis and Chemical Technology of Pharmaceutical Substances, Faculty of Pharmacy, Medical University of Lublin, 1 Al. Racławickie, 20 059 Lublin, Poland; 3 Department of Histology and Embryology, Centre of Biostructure Research, Medical University of Warsaw, 5 Chałubińskiego, 02 004 Warsaw, Poland; 4 Crystallochemistry Laboratory, Chemistry Department, Warsaw University, 1 Pasteura, 02 093 Warsaw, Poland

**Keywords:** Hydroxycoumarin, Microwave-assisted synthesis, Lipophilicity, Anti-cancer activity, X-ray structure

## Abstract

**Abstract:**

A new series of hydroxycoumarin derivatives has been synthesized using conventional synthesis. The syntheses were accelerated by microwave assistance. Yields in both cases were comparable (59–69 %). The structures were established by ^1^H and ^13^C NMR spectroscopy and high-resolution mass spectrometry. Five compounds (5-hydroxy-4,7-dimethylcoumarin, 6-acetyl-5-hydroxy-4,7-dimethylcoumarin, 4-(cyanomethoxy)chromen-2-one, 5-(cyanomethoxy)-4,7-dimethylchromen-2-one, and 6-acetyl-5-(cyanomethoxy)-4,7-dimethylchromen-2-one) were assayed for anti-cancer activity. For all presented coumarin derivatives, lipophilicity was measured using reversed-phase TLC in different eluent systems with standardization. In addition, the crystal structure of 6-acetyl-5-hydroxy-4,7-dimethylcoumarin has been solved by X-ray structure analysis of single crystals.

**Graphical abstract:**

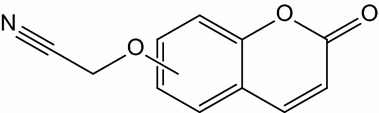

## Introduction

The cytotoxic activity of plant-derived hydroxycoumarins and their derivatives, synthetic analogues, has been reviewed in a number of studies [1]. The anti-tumor activity of 7-hydroxycoumarin (umbelliferone) and 4-hydroxycoumarin against human tumor cell lines, including prostate cancer, malignant melanoma, and metastatic renal cell carcinoma have been reported [2–4]. Geiparvarin, a naturally occurring compound bearing an umbelliferone residue has been shown to possess a significant inhibitory activity against a variety of cell lines including sarcoma 180, *Lewis* lung carcinoma, P-388 lymphotic leukemia, and *Walker* 256 carcinosarcoma [5, 6].

We have proved the importance of substitution on both phenolic group and *ortho*-position in the phenolic ring in a series of hydroxycoumarins. Introduction of acetyl group to *O*-alkyl derivatives of 4-methyl-7-hydroxycoumarin increased cytotoxicity and inhibited the growth of renal cancer 786-0, leukemia HL-60 (TB), leukemia CCRF-CEM, non-small- cell lung cancer HOP-92, and colon cancer HCC-2998 cell lines [7]. Cyanomethoxy derivatives of 7-hydroxycoumarin have been also shown as promising anti-tumor agents. 8-Acetyl-7-(cyanomethoxy)-4-methylcoumarin inhibited the growth of leukemia HL-60 (TB), K-562, RPMI-8226, non-small-cell lung cancer NCI-H522, and prostate cancer PC-3 cell lines [8].

Physicochemical properties have been widely applied to guide absorption, distribution, metabolism, and elimination (ADME) properties and pharmacological activities of discovery molecules, from small synthetic [9] to large natural or semisynthetic derivatives [10]. Lipophilicity has been one of the most used physicochemical properties useful in drug design, since it considerably influences bioavailability of compounds. Lipophilicity, as expressed by the logarithm of octanol/water partition coefficient log*P* (or distribution coefficient log*D* for ionizable compounds), plays an important role in ADME properties, as well as in the pharmacodynamic and toxicological profile of drugs [11, 12]. For lipophilicity assessment, partition chromatographic techniques and, in particular, reversed-phase HPLC offer several practical advantages compared to the traditional shake-flask method. These include speed, reproducibility, broader dynamic range, online detection, insensitivity to impurities or degradation products, and reduced sample handling and sample sizes [13, 14].

**Fig. 1:**
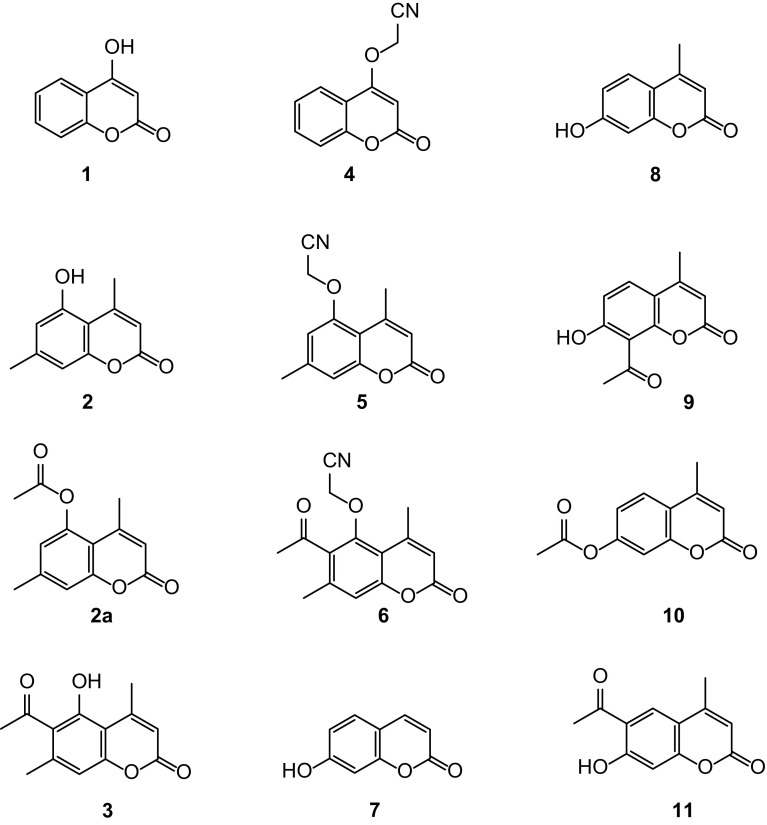
Structures of coumarins investigated

7-Hydroxycoumarins and 4-hydroxycoumarin derivatives have been the targets of our research [15–18]. A similar system, 5-hydroxycoumarin which is a promising target as a scaffold for new therapeutic agents was studied much less intensively, and the library of derivatives of 5-hydroxycoumarin is definitely less prominent. To fill the gap in this study, we present a new series of 4- and 5-cyanomethoxy derivatives of coumarin (Fig. 1) with an expected anti-cancer activity which have been synthesized using microwave irradiation and conventional synthesis and characterized by various methods.

The coumarins **2**, **3**, and **9**–**11** were resynthesized according to previously published papers [19–22]. The microwave-assisted synthesis has been used because it has many advantages over traditional methods, which are operational simplicity, good yields, short reaction times, and easy workup procedures [23]. For all presented compounds, lipophilic properties were determined experimentally and by calculating the partition coefficient by reversed-phase TLC technique in different solvent systems with measurement standardization. To complete the structural characterization, we also report the results of the X-ray crystallographic studies for 6-acetyl-5-hydroxy-4,7-dimethylcoumarin (**3**).

## Results and discussion

Here, we report a synthetic route for 4-(cyanomethoxy)chromen-2-one (**4**), 5-(cyanomethoxy)-4,7-dimethylchromen-2-one (**5**), and 6-acetyl-5-(cyanomethoxy)-4,7-dimethylchromen-2-one (**6**) synthesized from 4-hydroxycoumarin (**1**), 5-hydroxy-4,7-dimethylcoumarin (**2**), and 6-acetyl-5-hydroxy-4,7-dimethylcoumarin (**3**), respectively (Scheme 1). The reaction of coumarins **1**–**3** with the alkylating agent chloroacetonitrile under reflux in acetone, using anhydrous potassium carbonate as a base, was performed, resulting in three novel *O*-substituted cyanomethoxy coumarins **4**-**6**. Heating the 1-methyl-2-pyrrolidone solutions of substrates **1**–**3** with the alkylating agent in the presence of anhydrous potassium carbonate at the temperature 130–140 °C yielded the same products **4**–**6**. Reflux was applied for 16, 18, and for 24 h to obtain the compounds **4**, **5**, and **6** in acetone, and for 6, 10, and 12 h in 1-methyl-2-pyrrolidone at 130–140 °C, respectively.

**Figure:**
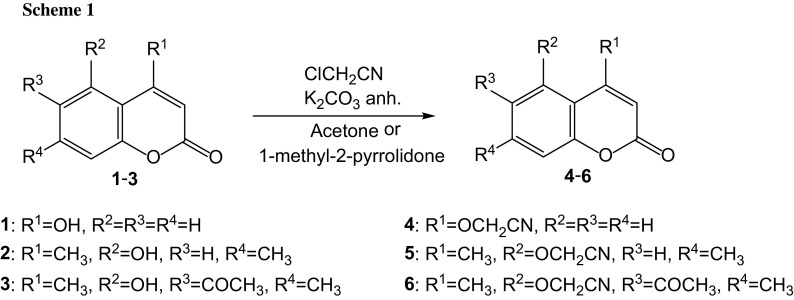

The *O*-alkylation reaction of starting coumarins **1**–**3** with the alkylating agent chloroacetonitrile under microwave irradiation afforded the products within few minutes. The syntheses were carried out under reflux in acetone or at the temperature 130–140 °C in 1-methyl-2-pyrrolidone, using anhydrous potassium carbonate as a base. The synthesis under microwave irradiation gave the same products **4**–**6**. The yields were a little higher or comparable with the yields of syntheses carried out in the conventional way. The major achievement of this procedure was the considerable reduction of reaction times: from 6 h to 12 min for compound **4**, from 10 h to 12 min for compound **5**, and from 12 h to 15 min for compound **6** (Table 1). Spectroscopic data (^1^H, ^13^C NMR, and mass) confirmed the structures of all products.

**Table 1:** The yield obtained and reaction time for compounds **4**, **5**, and **6**

Compound	Reaction time		Yield/%		
	Conventional/h	MW/min	Solvent	Conventional	MW
**4**	6	12	1-Methyl-2-pyrrolidone	59	60
**4**	16	12	Acetone	50	50
**5**	10	12	1-Methyl-2-pyrrolidone	69	70
**5**	18	12	Acetone	59	65
**6**	12	15	1-Methyl-2-pyrrolidone	60	67
**6**	24	15	Acetone	59	65

Coumarin **1** was obtained from Merck-Schuchardt, **7** and **8** were obtained from Sigma-Aldrich. Coumarins **2**, **2a**, **3**, and **9**–**11** were prepared according to previously published papers [19–22] (Fig. 1). 5-Hydroxy-4,7-dimethylcoumarin (**2**) was synthesized by Pechmann condensation of orcinol with ethyl acetoacetate in the presence of sulfuric acid [19]. Compound **2** was subjected to acetylation, thus obtaining 5-acetoxy-4,7-dimethylcoumarin (**2a**). From the latter product, 6-acetyl-5-hydroxy-4,7-dimethylcoumarin (**3**) was synthesized by means of Fries rearrangement [19]. 8-Acetyl-7-hydroxy-4-methylcoumarin (**9**) was obtained by heating under reflux a mixture of resorcinol, 2,6-dihydroxyacetophenone, ethyl acetoacetate, and catalytic amount of *p*-toluenesulfonic acid under reflux with azeotropic removal of water and ethanol [20].

7-Acetoxy-4-methylcoumarin (**10**) was synthesized by acetylation of compound **8** with acetic anhydride in pyridine in the presence of catalytic amount of (dimethylamino)pyridine (DMAP) [21]. 6-Acetyl-7-hydroxy-4-methylcoumarin (**11**) was obtained from 7-acetoxy-4-methylcoumarin (**10**) by heating with anhydrous aluminum trichloride [22]. The structures of coumarins **1**–**3** and **7**–**11** were established on the basis of their spectral data (^1^H, ^13^C NMR, and mass) and comparison of their melting points and spectral data with those reported in the literature.

The lipophilicity descriptors on analyzed compounds on which the entire classification has been performed are listed in Table 2. The lipophilicity descriptors on analyzed compounds on which the entire classification has been performed are listed in Table 3. The log*P* values of the tested compounds were determined by the series of standards with known log*P* value (Table 4) [24] using procedures described previously [18].

**Table 2:** The lipophilicity indices of samples obtained on RP-18 WF_254s_ stationary phase

Compound	**2**	**2a**	**3**	**4**	**5**	**6**	**7**	**8**	**9**	**10**	**11**
Methanol/water											
*S*	2.746	2.5335	3.2113	2.2036	2.677	2.3247	1.9022	2.0525	2.5967	2.3174	1.8855
*R*_M0_	1.8545	1.6267	2.4203	1.3375	1.7917	1.3412	0.9623	1.1629	1.8097	1.5769	0.9916
*R* ^2^	0.983	0.9931	0.9883	0.9715	0.9714	0.9875	0.9668	0.9688	0.9816	0.9871	0.9919
*φ*_0_	0.675	0.642	0.754	0.607	0.669	0.577	0.506	0.567	0.697	0.680	0.526
Acetonitrile/water											
*S*	3.2466	3.6418	3.706	2.8179	3.3589	3.7596	2.0876	2.5539	3.0614	3.506	2.9356
*R*_M0_	1.4191	1.7512	1.923	1.2332	1.6749	1.9451	0.7232	0.9747	1.4206	1.8452	1.248
*R* ^2^	0.9908	0.9943	0.9858	0.9901	0.9738	0.9937	0.9755	0.9897	0.9877	0.9968	0.9931
*φ*_0_	0.437	0.481	0.519	0.438	0.499	0.517	0.346	0.382	0.464	0.526	0.425
Dioxane/water											
*S*	3.3439	3.2936	4.1018	2.5467	3.2254	3.2502	1.8746	2.2314	3.2472	3.3341	2.5162
*R*_M0_	1.6138	1.5605	2.2559	1.1523	1.6055	1.6296	0.6676	0.912	1.6494	1.7279	1.0282
*R* ^2^	0.9738	0.9953	0.9827	0.9503	0.958	0.9938	0.9882	0.9929	0.9871	0.9924	0.9897
*φ*_0_	0.483	0.474	0.55	0.452	0.498	0.501	0.356	0.409	0.508	0.518	0.409
Isopropanol/water											
*S*	2.8574	3.8312	3.15	2.2371	2.3947	3.2079	1.6695	2.0405	2.6277	3.436	2.8074
*R*_M0_	1.2711	1.6259	1.5094	0.896	1.0398	1.2271	0.5162	0.7582	1.1726	1.5383	1.0153
*R* ^2^	0.9958	0.989	0.9919	0.9942	0.9864	0.9945	0.9719	0.997	0.9997	0.9904	0.9938
*φ*_0_	0.445	0.424	0.479	0.401	0.434	0.383	0.309	0.372	0.446	0.448	0.362

*φ*_0_ intercept of the trend line with abscissa axis (molar fraction of the polar solvent for which lipophilicity equals 0)

**Table 3:** The lipophilicity indices of standards obtained on RP-18 WF_254s_ stationary phase

Compound	Isatine	2-Nitrophenol	3,4-Dichloroaniline	3,4-Dichloroacetanilide	2,4-Dichloroaniline	Benzophenone	Diphenylamine	Diphenylmethane
Methanol/water								
*S*	1.4786	1.9676	3.2073	2.4827	2.7739	3.2945	2.9469	4.067
*R*_M0_	0.6009	1.0469	2.1151	1.7855	1.8895	2.3001	2.0947	3.1303
*R* ^2^	0.9915	0.9917	0.9882	0.9791	0.9939	0.9878	0.9903	0.9802
Acetonitrile/water								
*S*	2.0278	2.5429	3.2165	3.5551	3.3029	4.1335	4.1309	5.2957
*R*_M0_	0.7435	1.1246	1.7807	1.9258	1.8609	2.3558	2.4686	3.2971
*R* ^2^	0.928	0.9903	0.9922	0.9836	0.9773	0.9874	0.9953	0.991
Dioxane/water								
*S*	1.2132	2.2497	3.3499	3.0847	3.3036	3.6381	3.8375	5.0853
*R*_M0_	0.3418	1.0657	1.9357	1.7471	2.0135	2.2332	2.4348	3.4035
*R* ^2^	0.9105	0.9882	0.9958	0.9842	0.9985	0.9932	0.9974	0.9886
Isopropanol/water								
*S*	2.0065	2.9639	4.4484	4.1116	4.2876	4.4575	4.6873	6.2485
*R*_M0_	0.5885	1.2375	2.149	1.9521	2.1269	2.1794	2.3652	3.4449
*R* ^2^	0.8874	0.9895	0.9907	0.9845	0.9957	0.9861	0.9922	0.9762

**Table 4:** Log*P* values for used standards

Compound	Log*P* value
Isatine	0.83
2-Nitrophenol	1.79
3,4-Dichloroaniline	2.69
3,4-Dichloroacetanilide	2.96
2,4-Dichloroaniline	2.91
Benzophenone	3.18
Diphenylamine	3.5
Diphenylmethane	4.14

The regression correlation coefficients corresponding to Eq. 2 (see “Experimental”, “Lipophilicity”) are having a good linearity with *R*_M_ values through the molar fraction of organic modifier in mobile phase. The correlation coefficients (*R* ^2^) were between 0.9384 and 0.9997. The best results with the highest linearity were obtained for isopropanol/water mobile phase, which suggest that this system is appropriate for the further analysis of this group of compounds (Tables 3, 4).

The linear relationship between known log*P* values and experimental *R*_M0_ parameter for standards had been used for calculating experimental log*P* values for tested compounds. All results are shown in Tables 5 and 6.

**Table 5:** Experimental log*P* values for tested compounds **2**–**6**

Mobile phase	Log*P* = f(*R*_M0_)	**2**		**2a**		**3**		**4**		**5**		**6**	
		*R*_M0_	log*P*_exp_	*R*_M0_	log*P*_exp_	*R*_M0_	log*P*_exp_	*R*_M0_	log*P*_exp_	*R*_M0_	log*P*_exp_	*R*_M0_	log*P*_exp_
a	*y* = 1.2688*x* + 0.377	1.8545	2.73	1.6267	2.44	2.4203	3.45	1.3375	2.07	1.7917	2.65	1.3412	2.08
b	*y* = 1.2518*x* + 0.3158	1.4191	2.09	1.7512	2.51	1.923	2.72	1.2332	1.86	1.6749	2.41	1.9451	2.75
c	*y* = 1.1017*x* + 0.6602	1.6138	2.44	1.5605	2.38	2.2559	3.15	1.1523	1.93	1.6055	2.43	1.6296	2.46
d	*y* = 1.1922*x* + 0.3591	1.2711	1.87	1.6259	2.30	1.5094	2.16	0.896	1.43	1.0398	1.60	1.2271	1.82

*a* methanol/water, *b* acetonitrile/water, *c* dioxane/water, *d* isopropanol/water

**Table 6:** Experimental log*P* values for tested compounds **7**–**11**

Mobile phase	Log*P* = f(*R*_M0_)	**7**		**8**		**9**		**10**		**11**	
		*R*_M0_	Log*P*_exp_	*R*_M0_	Log*P*_exp_	*R*_M0_	Log*P*_exp_	*R*_M0_	Log*P*_exp_	*R*_M0_	Log*P*_exp_
a	*y* = 1.2688*x* + 0.377	0.9623	1.60	1.1629	1.85	1.8097	2.67	1.5769	2.38	0.9916	1.64
b	*y* = 1.2518*x* + 0.3158	0.7232	1.22	0.9747	1.54	1.4206	2.09	1.8452	2.63	1.248	1.88
c	*y* = 1.1017*x* + 0.6602	0.6676	1.40	0.912	1.66	1.6494	2.48	1.7279	2.56	1.0282	1.79
d	*y* = 1.1922*x* + 0.3591	0.5162	0.97	0.7582	1.26	1.1726	1.76	1.5383	2.19	1.0153	1.57

*a* methanol/water, *b* acetonitrile/water, *c* dioxane/water, *d* isopropanol/water

Analysis of the data obtained revealed that even small structural changes can produce substantial differences in lipophilicity of derivatives investigated, which is in accordance with previous studies on this class of compounds [25]. The lowest lipophilicity was found for compounds **7** (umbelliferone), **8** (7-hydroxy-4-methylcoumarin), **10** (7-acetoxy-4-methylcoumarin), and **4** (4-(cyanomethoxy)chromen-2-one). Such effect may be connected to the presence of the free hydroxyl groups (compounds **7** and **8**), acetoxy group (compound **11**), or cyanomethoxy group (compound **4**). Presence of both methyl and acetyl moieties (compounds **9** and **10**; **2** and **3**) increases lipophilicity. It is interesting that for 8-acetyl-7-hydroxycoumarin (**9**) and 6-acetyl-7-hydroxycoumarin (**10**) the lipophilicity differs by only ca. 0.2. It might suggest that substituents in position 6 would slightly increase lipophilicity when substituents in position 8 would lower it. In the group of 5-hydroxycoumarin derivatives, the presence of cyanomethoxy moiety lowers the lipophilicity in respect of compounds with free hydroxyl group (derivatives **2** and **5**; **3** and **6**).

5-Hydroxy-4,7-dimethylcoumarin (**2**), 6-acetyl-5-hydroxy-4,7-dimethylcoumarin (**3**), 4-(cyanomethoxy)chromen-2-one (**4**), 5-(cyanomethoxy)-4,7-dimethylchromen-2-one (**5**), and 6-acetyl-5-(cyanomethoxy)-4,7-dimethylchromen-2-one (**6**) were accepted for cytotoxicity testing. Initially they had been evaluated in the two-cell line panel consisting of the B16-F10 (melanoma) and DU145 (prostate). Compounds **2** and **3** were slightly active, while compounds **4**–**6** were considered inactive in the primary screen. Considering the cytotoxicity of the 4-, 5-, 7-cyanomethoxy derivatives of coumarins, only the introduction of cyanomethoxy group in 7-position seems to have pronounced effect [8]. Considering the lipophilicity in correlation with cytotoxic effect suggest that slight increase of lipophilicity (from 5-cyanomethoxy derivatives to 5-hydroxy derivatives lipophilicity differs by ca. 0.59), decreased the cytotoxicity of these compounds.

**Fig. 2:**
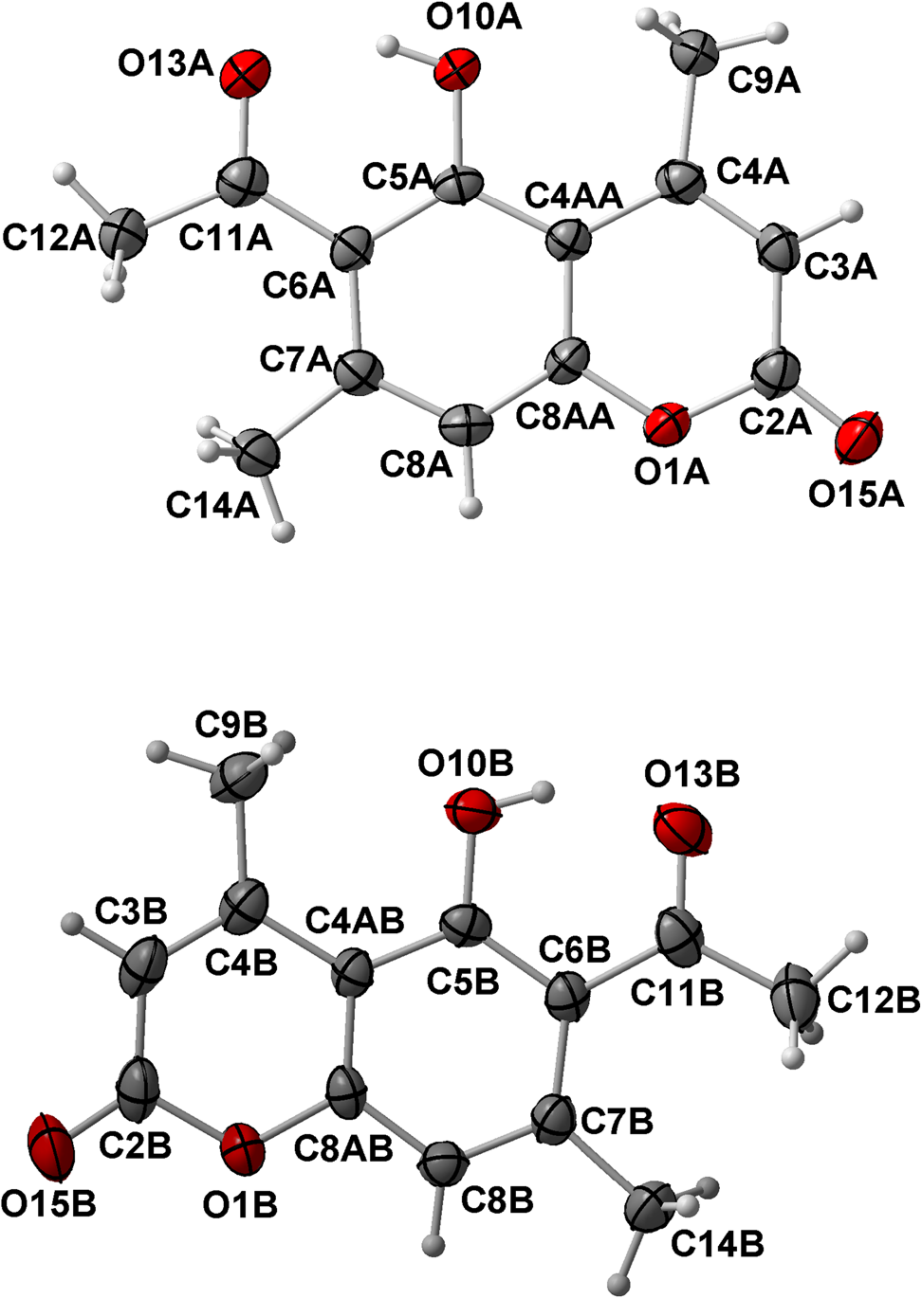
Numbering scheme and thermal ellipsoid at 50 % probability level for **3**

**Fig. 3:**
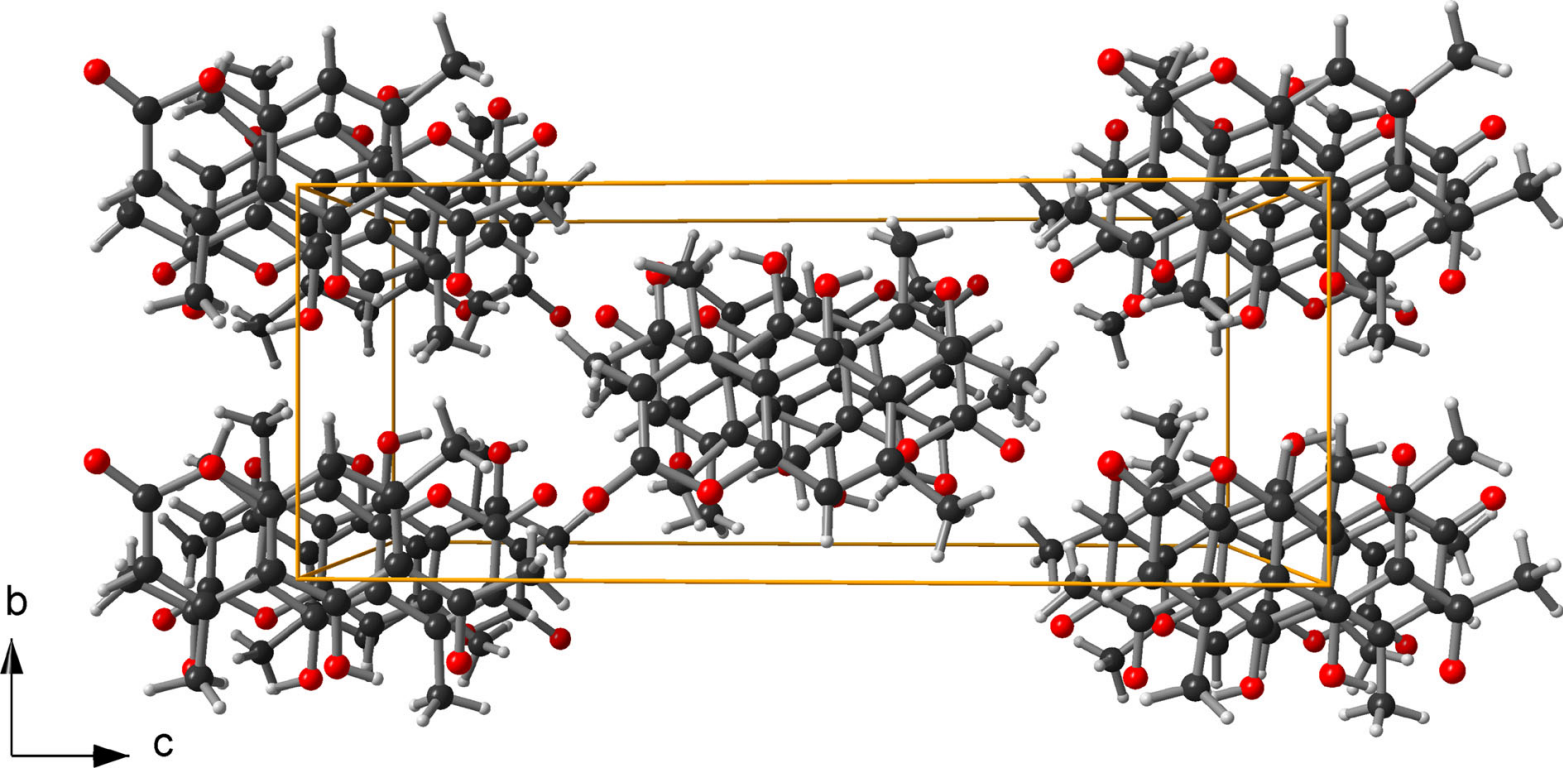
Packing diagram for **3**, view along [100]

**Fig. 4:**
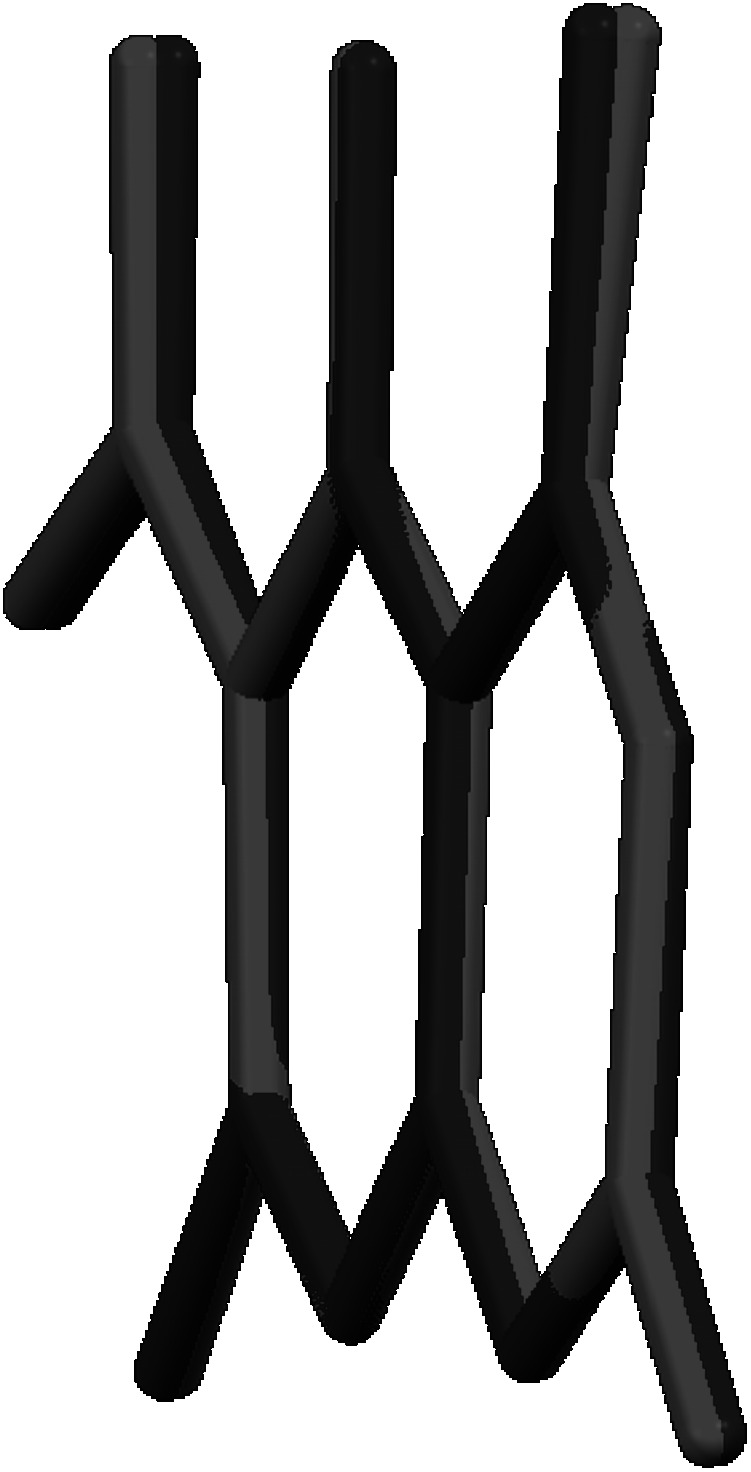
Overlay of chemically identical moieties A and B from crystal lattice of **3**

The molecular structure of 6-acetyl-5-hydroxy-4,7-dimethylcoumarin (**3**) in solid state was analyzed by single-crystal X-ray diffraction technique. Only for this compound we have obtained suitable crystals. Compound **3** crystallizes in the P2_1_/c space group. Crystal data and structure refinement parameters for **3** are collected in Table 7. Thermal ellipsoid plot and packing diagrams are presented in Figs. 2 and 3, respectively. The independent part of the crystal lattice of presented compound consists of two chemical moieties numbered A and B. While the B molecule is almost flat in the A one chromene skeleton is slightly twisted along the longer axis of the molecule. This is visible in Fig. 4 presenting overlay of molecules A and B calculated in Mercury program [26].

**Table 7:** Crystal data and structure refinement parameters for **3**

Identification code	B1
Chemical formula	C_13_H_12_O_4_
Formula weight	232.23
Temperature/K	100(2)
Wavelength/Å	0.71073
Crystal size/mm^3^	0.080 × 0.160 × 0.250
Crystal system	Monoclinic
Space group	P2_1_/c
Unit cell dimensions	
*a*/Å	14.0681(8)
*b*/Å	7.5749(4)
*c*/Å	9.6802(11)
*β*/°	95.465(2)
Volume/Å^3^	2,087.7(2)
*Z*	8
Density (calculated)/g cm^−3^	1.478
Absorption coefficient/mm^−1^	0.110
*F*(000)	976
*θ* range for data collection/°	2.42–26.50
Index ranges	−17 ≤ h ≤ 17, −9 ≤ *k* ≤ 9, −24 ≤ l ≤ 24
Reflections collected	25,586
Independent reflections	4,330 [*R*(int) = 0.0435]
Data completeness/%	100.0
Absorption correction	Multi-scan
*T*_max_/*T*_min_	0.9913/0.9730
Refinement method	Full-matrix least-squares on *F* ^2^
Data/restraints/parameters	4,330/0/315
Goodness-of-fit on *F* ^2^	1.121
Final *R* indices	3,378 data; *I* > 2*σ*(*I*)
	*R*1 = 0.0512, *wR*2 = 0.1251
	All data
	*R*1 = 0.0695, *wR*2 = 0.1350
Largest diff. peak and hole/e Å^−3^	0.374 and −0.206

**Fig. 5:**
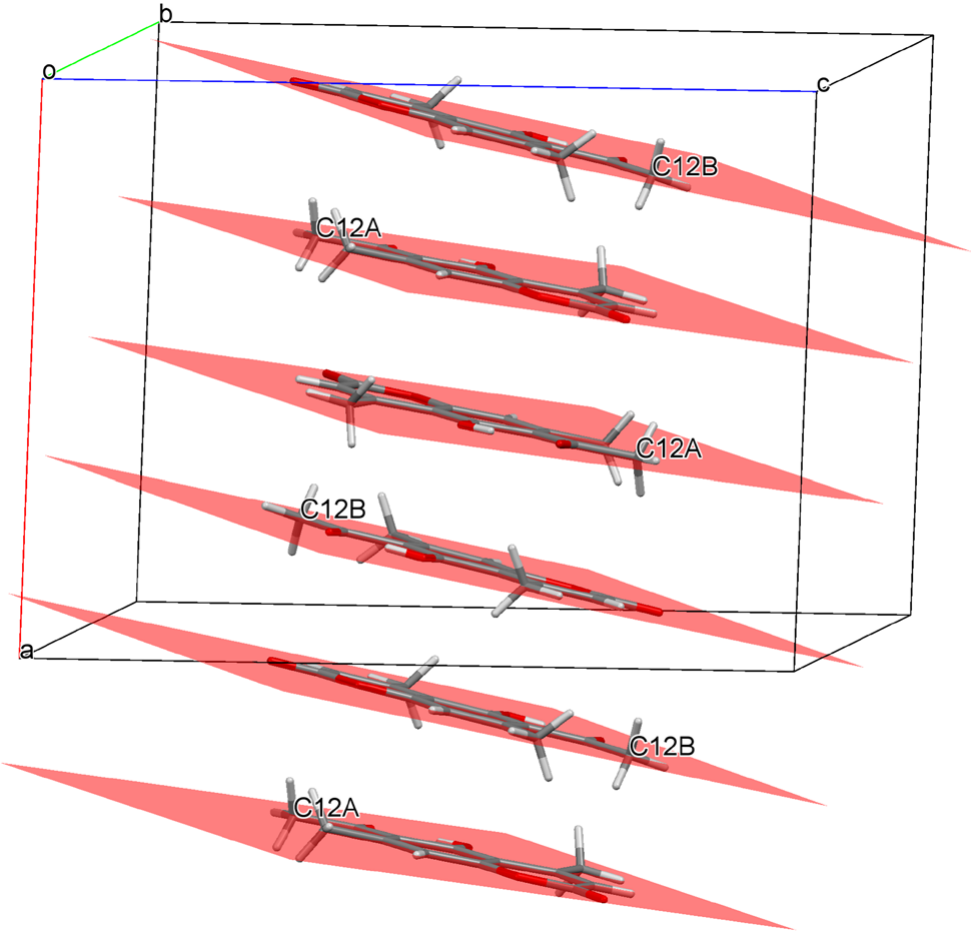
Stacking of molecules in the crystal lattice of **3**

In both moieties methyl group (C9) is visibly deflected from hydroxyl group. The angle C4A-C4-C9 is comparable in both cases and yields 123.3(2)° and 122.1(2)° in A and B molecules, respectively. There are no strong intermolecular interactions in the crystal structure of **3**. This is due to the fact that OH group (O10) is engaged in intramolecular hydrogen bond with carbonyl oxygen atom (O13). Distance O…O is equal to 2.435(2) Å and 2.430(2) Å in molecules A and B, respectively. In the crystal lattice molecules are forming stacks along [100] direction. In each stack molecules A and B are located alternately and average intermolecular distance is equal ca. 3.4 Å (Fig. 5).

## Experimental

Reagents were purchased from Aldrich or Merck, of the higher grade available and used without further purification. Solvents were used as received from commercial suppliers, and no further attempts were made to purify or dry them. Melting points were determined with Electrothermal 9001 Digital Melting Point apparatus (Electrothermal, Essex, UK). Microwave oven Plazmatronika 1,000 W equipped with a single-mode cavity suitable for the microscale synthesis and microwave choked outlet connected to external condenser set to 30 % power was used (http://www.plazmatronika.com.pl). High-resolution mass spectra were recorded on Quattro LCT (TOF). ^1^H NMR, ^13^C NMR, HSQC, and HMBC spectra in solution were recorded at 25 °C with a Varian Unity plus-500 spectrometer and standard Varian software was employed (Varian, Inc., Palo Alto, CA, USA). The calculated shielding constants were used as an aid in an assignment of resonances of ^13^C atoms. The CPHF-GIAO approach for the NMR shielding constants calculations using Gaussian 09 program was employed [26]. Chemical shifts *δ*/ppm were referenced to TMS. TLC was carried out using Kieselgel 60 F_254_ sheets (Merck, Darmstadt, Germany); spots were visualized by UV (254 and 365 nm). Kieselgel 60 was used for column chromatography.

### General procedure for the conventional syntheses

A mixture of appropriate coumarin **1**–**3** (7.5 mmol) and 0.5 cm^3^ chloroacetonitrile (8.25 mmol) were dissolved in 10 cm^3^ acetone or 1-methyl-2-pyrrolidone, and 3.1 g anhydrous K_2_CO_3_ (22.5 mmol) was added to this solution. The mixture was refluxed (acetone) or heated at the temperature 130–140 °C (1-methyl-2-pyrrolidone) and monitored by TLC on silica-gel plates (eluent CHCl_3_–MeOH 10:0.25). After completion of the reactions as indicated by TLC, the mixture was poured into the flask with 50 cm^3^ water and ice and stirred for 30 min. The precipitate was filtered out and dried. The analytical samples were crystallized from ethanol.

### General procedure for the microwave-assisted syntheses

A mixture of appropriate coumarin **1**–**3** (7.5 mmol) and 0.5 cm^3^ chloroacetonitrile (8.25 mmol) and 3.1 g K_2_CO_3_ (22.5 mmol) were placed in a microwave flask, and 7 cm^3^ of acetone or 1-methyl-2-pyrrolidone was added. The mixture was refluxed (acetone) or heated at the temperature 130–140 °C (1-methyl-2-pyrrolidone) in the monomode microwave oven (300 W) and monitored by TLC on silica-gel plates (eluent CHCl_3_–MeOH 10:0.25). Four cycles were needed to obtain 4-(cyanomethoxy)chromen-2-one (**4**), 5-(cyanomethoxy)-4,7-dimethylchromen-2-one (**5**), and 6-acetyl-5-(cyanomethoxy)-4,7-dimethylchromen-2-one (**6**). Heating time for each cycle was 3 min. After completion of the reactions as indicated by TLC, 100 cm^3^ ice water was added to the reaction mixture and stirred for 30 min. The precipitate was filtered out and dried. The analytical samples were crystallized from ethanol.

#### *4*-*(Cyanomethoxy)chromen*-*2*-*one* (**4**, C_11_H_7_NO_3_)

Yield: (a) 1-methyl-2-pyrrolidone, conventional, 59 %, (b) acetone, conventional, 50 %, (c) 1-methyl-2-pyrrolidone, MW, 60 %, (d) acetone, MW, 50 %; m.p.: 204 °C; *R*_f_ = 0.68; ^1^H NMR (300 MHz, CDCl_3_): *δ* = 7.79 (d, *J* = 9.6 Hz, 1H, H-5), 7.61 (dd, *J* = 8.7 Hz, 1H, H-7), 7.34 (m, 2H, H-6, H-8), 5.79 (s, 1H, H-3), 4.95 (s, 2H, H-9) ppm; ^13^C NMR (125 MHz, CDCl_3_): *δ* = 53.6 (C-9), 92.2 (C-3), 113.01 (C-10), 114.6 (C-4a), 117.2 (C-8), 123.0 (C-5), 124.6 (C-6), 133.5 (C-7), 153.6 (C-8a), 161.6 (C-2), 163.6 (C-4) ppm; MS (TOF, ES+): [M+Na]^+^ calcd for C_11_H_7_NNaO_3_ 224.0328, found 224.0324.

#### *5*-*(Cyanomethoxy)*-*4,7*-*dimethylchromen*-*2*-*one* (**5**, C_13_H_11_NO_3_)

Yield: (a) 1-methyl-2-pyrrolidone, conventional, 69 %, (b) acetone, conventional, 59 %, (c) 1-methyl-2-pyrrolidone, MW, 70 %, (d) acetone, MW, 65 %; m.p.: 214–216 °C; *R*_f_ = 0.79; ^1^H NMR (300 MHz, CDCl_3_): *δ* = 6.89 (s, 1H, H-8), 6.58 (s, 1H, H-6), 6.12 (s, 1H, H-3), 4.87 (s, 2H, H-9), 2.56 (d, *J* = 1.2 Hz, 3H, H-4), 2.44 (s, 1H, H-7) ppm; ^13^C NMR (125 MHz, CDCl_3_): *δ* = 22.2 (C-10), 24.4 (C-11), 54.0 (C-9), 108.5 (C-8), 109.1 (C-3), 113.0 (C-6), 114.5 (C-12), 115.1 (C-4a), 143.4 (C-7), 152.9 (C-8a), 154.6 (C-4), 155.6 (C-5), 160.5 (C-2) ppm; MS (TOF, ES +): [M + Na]^+^ calcd for C_13_H_11_NNaO_3_ 252.0632, found 252.0637.

#### *6*-*Acetyl*-*5*-*(cyanomethoxy)*-*4,7*-*dimethylchromen*-*2*-*one* (**6**, C_15_H_13_NO_4_)

Yield: (a) 1-methyl-2-pyrrolidone, conventional, 60 %, (b) acetone, conventional, 59 %, (c) 1-methyl-2-pyrrolidone, MW, 67 %, (d) acetone, MW, 65 %; m.p.: 283–284 °C; *R*_f_ = 0.59; ^1^H NMR (300 MHz, CDCl_3_): *δ* = 7.09 (d, *J* = 0.6 Hz, 1H, H-8), 6.25 (d, 1H, *J* = 1.5 Hz, H-3), 4.63 (s, 2H, H-11), 2.64 (s, 3H, H-10), 2.56 (s, 3H, H-9), 2.32 (s, 3H, H-4) ppm; ^13^C NMR (125 MHz, CDCl_3_): *δ* = 19.4 (C-9), 22.9 (C-12), 32.8 (C-10), 61.3 (C-11), 112.9 (C-4a), 114.1 (C-8), 117.2 (C-3), 117.4 (C-6), 133.6 (C-11), 139.3 (C-7), 150.9 (C-8a), 154.8 (C-4), 154.7 (C-5), 159.5 (C-2), 204.3 (C-13) ppm; MS (TOF, ES+): [M+Na]^+^ calcd for C_15_H_13_NNaO_4_ 294.0742, found 294.0742.

#### Lipophilicity

Standards: isatine, benzoic acid, 2-nitrophenol, diphenylamine were purchased from POCH (Avantor Performance Materials Poland S.A.), 3,4-dichloroaniline, 2,4-dichloroaniline, benzophenone from MERCK, diphenylmethane from Koch-Light. 3,4-Dichloroacetanilide was synthesized from 3,4-dichloroaniline and acetic anhydride. All reagents and chemicals were analytical purity grade.

#### Preparation of samples and standard solutions

Tested compounds (1 mg) were weighed in Eppendorf tubes and directly dissolved in 1 cm^3^ of methanol. All standards for TLC were prepared by solving 1 mg of substance in 1 cm^3^ of methanol.

#### Chromatographic conditions

TLC analysis was performed on HPTLC silica gel, 10 × 10 cm, RP-18 WF_254s_ glass plates (Merck, Germany). Samples and standards solutions were applied as a 1-mm spot onto plate using 0.5-mm^3^ thin glass capillary tube (Camag, Switzerland). The distance between each application was 10 and 10 mm distance from low edge of the plate. The plate was developed to a distance of 80 mm in a TLC horizontal chamber (Chromdes, Poland) previously saturated with appropriate solvent for 10 min in 20 °C.

Mobile phases were mixtures of acetonitrile (*φ* = 0.25/0.30/0.35/0.40/0.45/0.50/0.55/0.60), dioxane (*φ* = 0.30/0.35/0.40/0.45/0.50/0.55/0.60), methanol (*φ* = 0.4/0.45/0.5/0.55/0.6/0.65/0.7/0.75/0.8), isopropanol (*φ* = 0.3/0.35/0.4/0.45/0.5/0.55) with water. All solvents used for preparing mobile phase (acetonitrile, dioxane, isopropanol, methanol) were purchased from POCH (Avantor Performance Materials Poland S.A.). The solvents were of HPLC purity grade. The water for chromatography was produced within the laboratory by means of a MILLIPORE, MILLI-Q INTEGRAL 3 distillation system and used during the experiments.

#### TLC-image analysis method

Air-dried in room temperature HPTLC plate was visualized under UV-light at 254 and 366 nm (TLC-Visualiser, Camag) and saved as lossless JPEG file. The image was opened with winCATS software (Camag, Switzerland) and the *R*_F_ parameter was calculated.

#### Chromatographic parameters

*R*_M_ values for tested compounds (Table 2) and standards (Table 3) were calculated from the experimental *R*_F_ by the use of the Eq. 1.

1 $$R_{\text{M}} \, = \, \,{ \log }\left( { 1 { }/R_{\text{F}} \, - \, \, 1} \right)$$

The calculated *R*_M_ values were extrapolated to 0 % organic modifier concentration (*R*_M0_) by use of Eq. 2:

2 $$R_{\text{M}} \, = \,R_{{{\text{M}}0}} \, - \,S\varphi ,$$

where *φ* (molar fraction) describes concentration of organic modifier in mobile phase, *S* is a slope of the trend line and *R*_M0_ is extrapolated value of the lipophilicity for 100 % of water.

### Cytotoxicity against cancer cell lines

#### Reagents

Synthesized substances were dissolved in DMSO (Sigma-Aldrich) to obtain 20 mM stock solutions and kept in 4 °C prior to use.

#### Cell culture

The human prostate cancer cells DU145 and mouse melanoma cells B16F10 were maintained in humidified incubator containing 5 % CO_2_ at 37 °C. DU145 cells were cultured in RPMI medium and B16F10 were cultured in DMEM medium. The media were supplemented with 10 % of fetal calf serum (FCS) and 1 % of antibiotic antimycotic (Sigma). Cells were passaged every 2–3 days. All cell lines were obtained from ATCC (The Global Bioresource Center).

#### Cytotoxicity assays

PrestoBlue (test based on resazurin) was used for analysis cytotoxicity/cytostatic effects. DU145 and B16F10 cells were seeded on 96-well plate (10 × 10^3^ cells per well). Examined substances were added to culture after overnight cell incubation. Two concentrations of substances (10 and 100 μM) were used. Control cells and control cells with solvent (DMSO) were applied. After 48 h PrestoBlue test was performed.

### Crystallography

The X-ray measurement of **3** was performed at 100(2) K on a Bruker D8 Venture Photon100 diffractometer equipped with a TRIUMPH monochromator and a MoKα fine focus sealed tube (*λ* = 0.71073 Å). A total of 660 frames were collected with Bruker APEX2 program [27]. The frames were integrated with the Bruker SAINT software package [28] using a narrow-frame algorithm. The integration of the data using a monoclinic unit cell yielded a total of 25,586 reflections to a maximum *θ* angle of 26.50° (0.80 Å resolution), of which 4,330 were independent (average redundancy 5.909, completeness = 100.0 %, *R*_int_ = 4.35 %, *R*_sig_ = 2.61 %) and 3,378 (78.01 %) were greater than 2*σ*(*F* ^2^). The final cell constants of *a* = 14.0681(8) Å, *b* = 7.5749(4) Å, *c* = 19.6802(11) Å, *β* = 95.465(2)°, volume = 2,087.7(2) Å^3^, are based upon the refinement of the XYZ-centroids of 8,583 reflections above 20 *σ*(*I*) with 4.842° < 2*θ* < 54.16°. Data were corrected for absorption effects using the multi-scan method (SADABS) [29]. The ratio of minimum to maximum apparent transmission was 0.837. The calculated minimum and maximum transmission coefficients (based on crystal size) are 0.9730 and 0.9913.

The structure was solved and refined using SHELXTL software package [30, 31] using the space group P 1 21/c 1, with *Z* = 8 for the formula unit C_13_H_12_O_4_. The final anisotropic full-matrix least-squares refinement on *F* ^2^ with 315 variables converged at *R*1 = 5.12 %, for the observed data and *wR*2 = 13.50 % for all data. The goodness-of-fit was 1.121. The largest peak in the final difference electron density synthesis was 0.374 e/Å^3^ and the largest hole was −0.206 e/Å^3^ with an RMS deviation of 0.060 e/Å^3^. On the basis of the final model, the calculated density was 1.478 g/cm^3^ and *F*(000) 976 e.

The non-hydrogen atoms were refined anisotropically. All hydrogen atoms were placed in calculated positions and refined within the riding model. In addition CH_3_ and OH groups were free to rotate along C–C and C–O bonds, respectively. The temperature factors of hydrogen atoms were not refined and were set to be equal to either 1.2 or 1.5 times larger than *U*_eq_ of the corresponding heavy atom. The atomic scattering factors were taken from the International Tables [32]. Molecular graphics were prepared using program Diamond 2.1 [33]. Thermal ellipsoids parameters are presented at 50 % probability level.
